# Measuring Implicit European and Mediterranean Landscape Identity: A Tool Proposal

**DOI:** 10.3389/fpsyg.2016.01259

**Published:** 2016-09-02

**Authors:** Ferdinando Fornara, Francesco Dentale, Renato Troffa, Simona Piras

**Affiliations:** ^1^Department of Education, Psychology, Philosophy, University of CagliariSardinia, Italy; ^2^Centro Interuniversitario di Ricerca in Psicologia Ambientale – Interuniversity Research Center in Environmental PsychologyRome, Italy; ^3^Department of Psychology, Sapienza University of RomeRome, Italy

**Keywords:** landscape identity, place identity, European identification, Mediterranean identification, implicit association test

## Abstract

This study presents a tool – the Landscape Identity Implicit Association Test (LI-IAT) – devoted to measure the implicit identification with European and Mediterranean landscapes. To this aim, a series of prototypical landscapes was selected as stimulus, following an accurate multi-step procedure. Participants (*N* = 174), recruited in two Italian cities, performed two LI-IATs devoted to assess their identification with European vs. Not-European and Mediterranean vs. Not-Mediterranean prototypical landscapes. Psychometric properties and criterion validity of these measures were investigated. Two self-report measures, assessing, respectively, European and Mediterranean place identity and pleasantness of the target landscapes, were also administered. Results showed: (1) an adequate level of internal consistency for both LI-IATs; (2) a higher identification with European and Mediterranean landscapes than, respectively, with Not-European and Not-Mediterranean ones; and (3) a significant positive relationship between the European and Mediterranean LI-IATs and the corresponding place identity scores, also when pleasantness of landscapes was controlled for. Overall, these findings provide a first evidence supporting the reliability and criterion validity of the European and Mediterranean LI-IATs.

## Introduction

### Identification with Landscapes

The psychological responses to the landscapes represent an issue widely investigated in the literature, with a particular focus on the patterns of affective and esthetic evaluation (see Kaplan and Kaplan, 1989; Kaltenborn and Bjerke, 2002). This perspective taps the so-called “subjective” assessment of landscapes, which in fact concerns the perceptual side, and is contrasted to the “objective” assessment of landscapes, which addresses their capacity to provide a set of benefits for the ecosystem (see Aretano et al., 2013). Within the “subjective” domain, that is the focus of this paper, a substantially unexplored field is the identitarian meaning of landscapes, which refers to their image as a potential source of (shared) identification for a given community. In this regard, Hagerhall (2001) pointed out that the Swedish traditional cultural landscape, including pastures and meadows, has assumed a prominent position in the national history, being also frequently portrayed in Swedish literature and art. Hence, it is thus not surprising that this kind of images is used for advertising goals (Hagerhall, 2001), in order to convey messages that should connect the positive connotation evoked by the landscape to the self-identity of persons who identify with it.

The present study proposes a tool for measuring the implicit identification with those landscapes referring to broad geographical scales represented by the European continent and the Mediterranean area. In fact, we assumed that those persons living in the European and Mediterranean zone could consider these two targets as meaningful places.

Landscape identity is here conceived as a specific component of place identity, that is a concept originally developed by Proshansky et al. (1983, p. 60), who defined it as “a set of memories, conceptions, interpretations, and feelings related to a specific physical setting.” In other words, place identity is conceived as a component of the self-concept referring to the place one belongs to Hernández et al. (2007) and Lewicka (2010). More specifically, this construct concerns “those dimensions of the self that define the individuals’ personal identity in relation to the physical environment, by means of a complex pattern of conscious and unconscious ideas, beliefs, preferences, feelings, values, goals, behavioral tendencies, and skills relevant to this environment” (Proshansky, 1978, p. 155). Landscapes may be considered as important sources of identity, as they embody not only environmental identification processes but also social meanings (Twigger-Ross et al., 2003). In this view, for instance, on the basis of the ingroup bias effect postulated by the Social Identity Theory (SIT: Tajfel and Turner, 1986), the membership in social groups that are located in a specific place (i.e., a setting delimited by spatial-physical boundaries) may in fact represent a support for the maintenance of a positive place identity (Carrus et al., 2014).

Different conceptual paths have been followed in order to investigate the relationship between place and identity patterns. Some studies focused primarily on the identification with a place and on the attributes that give a distinctive place identity to the residents (e.g., Schneider, 1987; Uzzell et al., 2002). In other studies, the identity of the place is conceived as a facet of personal and social identity (i.e., a “localized” social identity) that stems from processes of identification, social cohesion, and residential satisfaction (Valera and Pol, 1994). In this vein, the extent to which individuals identify themselves with a prototypical landscape is expected to be related to the degree of their community identification. Place identity patterns have typically been studied at different levels of scale, such as local (e.g., urban identity, see Lalli, 1992; Valera et al., 1998; Bonnes et al., 2011), regional (e.g., Abrams and Emler, 1992; Carrus et al., 2005), and national (e.g., Smith, 1992) scale. Instead, here we focus on a supranational level, given that our target places are, respectively, “Europe” and “Mediterranean,” both representing socially constructed entities which include geographical, social, and political meanings.

In fact, we acknowledge that images of our city or specific sites of our country are part of our local or national identity, but what about landscapes concerning identity targets at a broader scale in geographical terms, such as for example Europe and Mediterranean? Do prototypical images of Europe and the Mediterranean, typically not so much salient in our daily life, play a role in enhancing identification patterns at a broader geo-cultural scale?

### European and Mediterranean Identities

Europe and Mediterranean are geographical notions that present different characters, since the latter is outlined by marked physical boundaries (i.e., those countries “touched” by the Mediterranean Sea) whereas the former is a complex entity in continuous change (Guarracino, 2007). Both notions include historical, political, social, and economic features.

At the European level, policy actions have been conducted from past centuries, since from the times of the Roman Empire (see Dinan, 2004), in order to build up supranational bodies and overpass economic and political barriers. The increased diffusion of the European currency and the continuous expansion of the political union represented a cornerstone of this process. Nevertheless, the feeling to be a European citizen as well as the development of a European identity seem rather far to be achieved, as showed by the results of anti-Euro/anti-EU parties at the last elections for the UE Parliament in the 2014 as well as in the more recent national and local elections in various EU countries. Indeed, the development of a European identity should be a key element in order to overcome cultural, economic, social, political, linguistic, and religious barriers between EU nations that historically have determined conflicts and wars. As stated by Habermas (1998), who invokes “unity in diversity,” the unique variety of landscapes in Europe – reflecting a common geography, history and culture – may be useful to strengthen the European identity, as a way for stressing common characteristics but respecting differences among nations. According to Stanners and Bourdeau (1995), despite the dramatic socio-economic changes that have accompanied the wave of industrialization and urbanization of the last century in Europe, most differences remained, giving a distinctive character to countries, regions, and municipalities. The first environmental assessment, carried out by the European Agency for the Environment, showed that the European continent is characterized by a great variety of landscapes, which have been shaped through a long and intense interaction between biophysical and cultural factors (European Environment Agency, 2011). The various environmental conditions, in terms of slope of the soil, topography, and climate, as well as the different religious and ideological traditions, give form to the wide variety of European landscapes, which thus have become an essential feature of cultural identities. In this sense, landscape refers to the relationship between people and place, including how natural and cultural facets of the environment interact together, and how people perceive such facets (European Landscape Convention, 2000).

As regards the Mediterranean notion, it refers to a common environment that merges together those individuals, living in different cultures, bordering on the geographic region of the Mediterranean Sea (Fabre, 1998; Aubenas and Benasayag, 2002). It represents a unique environment, which generated and fed different cultures over the millennia (Petruzzellis and Craig, 2014), and possesses a distinctive trait that goes beyond the concepts of regionalism, local roots, ethnocentrism, and nationalism (Cassano, 1998). In this regard, the Mediterranean identity can be seen as the result of a millennial stratification of diasporas that has produced mixed cultures and habits, and the current migration from the south to the north is a sort of continuation of ancient movements (Tarrius, 1992). To better understand the relationship between the roots of Mediterranean cultures, Petruzzellis and Craig (2014) carried out a comparative analysis of identity patterns by interviewing inhabitants of three countries bordering the Mediterranean, that is France, Italy, and Spain. Respondents revealed a common identity related to their proximity to the Mediterranean Sea, suggesting the presence of bonds that transcend political boundaries. The Mediterranean essence, shaped by the Mediterranean Sea, is represented in residents’ narratives by mild climate, physical traits of the inhabitants (e.g., hair or skin color), slow and relaxed lifestyle, warmth, bright colors, symbols such as the kitchen or the dinner table, and crops like olive trees and vineyards. In Spanish and French respondents the landscape representations of the Atlantic Ocean and of the Mediterranean Sea were different: in fact, the first is perceived as dark, strong, wavy, and menacing, whereas the second is seen as bright, calm, quiet, and cozy. These different landscape images recall the distinction highlighted by Cassano (1998), who claimed that the Mediterranean is not an “empty” sea as the Ocean, but rather a basin that interconnects different lands, thus making possible a rich network of relationships (Petruzzellis and Craig, 2014). Not surprisingly, Italian and Spanish interviewees showed in their words more evident Mediterranean roots than the French ones, probably for the closeness of the latter, both geographically and culturally, to the Central and Northern Europe countries.

In conclusion of this literature review on European and Mediterranean identities connected to landscapes, it is to highlight that, despite the supposed role of such identities for creating and shaping positive psychological responses with important community implications, there is a substantial lack of empirical research on the European and the Mediterranean identity. This was one of the drivers of our study.

### Promoting the Use of Implicit Measures of Place Identity and Related Constructs

An array of tools has been proposed to measure constructs concerning the identification with places and other environmental targets, such as place identity (e.g., Hernández et al., 2007), place attachment (e.g., Lewicka, 2008; Fornara et al., 2010), and identification with nature (e.g., Schultz, 2001; Clayton, 2003; Mayer and Frantz, 2004).

As well as other self-report measures, these tools are exposed to two typical weaknesses: the proneness to response biases due to impression management effects (Paulhus, 1991; Vecchione et al., 2014) and the impossibility to tap all construct-related information using introspection (Nisbett and Wilson, 1977), for both self-deception (Paulhus, 1991) and cognitive factors (Hofmann et al., 2005; Dentale et al., 2012). Recently, mono and dual models of social cognition, which distinguish implicit and explicit processes, were developed and empirically supported (see Gawronski and Payne, 2010, for a review). Interestingly, they provided an important conceptual framework to address the factors that may threaten the validity of self-report measures, such as impression management responding and introspective limits. Following this line of reasoning, many attempts have been conducted to develop reliable and valid implicit measures of psychological constructs. Among them, the most popular and tested is the Implicit Association Test (IAT), first developed by Greenwald et al. (1998) and successively used in many areas of psychological research, such as attitudes, stereotypes, and self-esteem. Classical IAT is a time reaction task that permits to assess the degree to which subjects associate two target categories (e.g., Blacks vs. Whites) with two target attributes (e.g., Positive vs. Negative).

Even though IAT procedures have been typically used in many social domains (Nosek et al., 2005), there are also a few examples in the environmental field, with reference to the energy sources (Truelove et al., 2014) and to the identification with nature (Schultz et al., 2004). In the second case, which is closer to our study object, Schultz et al. (2004) developed the “IAT-Nature” in a study about the relationship between implicit and explicit connectedness with nature. It was found that the implicit attitude has a positive correlation with the biospheric concern, and a negative correlation with the egoistic concern. Similar results about the relationship between implicit and explicit attitudes toward nature emerged in more recent studies (Schultz and Tabanico, 2007; Brügger et al., 2011; Olivos and Aragonés, 2013).

### Objective and Hypotheses

This study aims at evaluating the psychometric properties and the criterion validity of two IATs devoted to measure European and Mediterranean Landscape Identity. In order to do this, different kinds of scenarios, reflecting a supranational geopolitical scale, were used as stimuli for the attribute categories (i.e., European *vs*. Not European for the first IAT; Mediterranean *vs*. Not Mediterranean for the second IAT), whereas a series of words were used as stimuli for the target categories (Self *vs*. Other). In particular, four types of prototypical landscapes were selected, using a three steps procedure (see next section). These Landscape Identity IATs (LI-IATs) allow to measure the degree to which European and Mediterranean landscapes are automatically associated with the self-concept.

Four specific hypotheses were tested to evaluate the criterion validity of the LI-IATs.

Considering that all participants are resident in a geographic area that is either European and Mediterranean, it was expected that:

(H1) The self-concept is more automatically associated with European landscapes rather than with Not-European ones;
(H2) The self-concept is more automatically associated with Mediterranean landscapes rather than with Not-Mediterranean ones.
Both European and Mediterranean LI-IATs were hypothesized to be significantly correlated with a closer criterion. Thus, it was expected that:
(H3) The automatic identification with European Landscapes is significantly related to a self-rating measure of European place identity;
(H4) The automatic identification with Mediterranean Landscapes is significantly related to a self-rating measure of Mediterranean place identity.

## Materials and Methods

One hundred and seventy four participants, 56% females and 44% males, with a mean age of 32.94 (*SD* = 9.57), were recruited in Rome and Cagliari, which are the main urban agglomerations of, respectively, Lazio and Sardinia, i.e., two Italian Regions bordering on the Mediterranean sea. Concerning the education level, 5% of the sample attended the Junior High School, 36% the Senior High School, and 59% had a Degree.

Participants had to perform two IAT tasks, one devoted to measure the European LI and the other Mediterranean LI, including both images and words as stimuli. After the completion of the IATs, participants filled in a questionnaire including an adaptation of the self-categorization tool consisting in two items, one including the concepts of Self and Europe, the other including the concepts of Self and Mediterranean. Successively, participants had to assess on a self-report scale the pleasantness of all images presented in the IATs.

### Landscape Identity IATs (LI-IATs)

**FIGURE 1:**
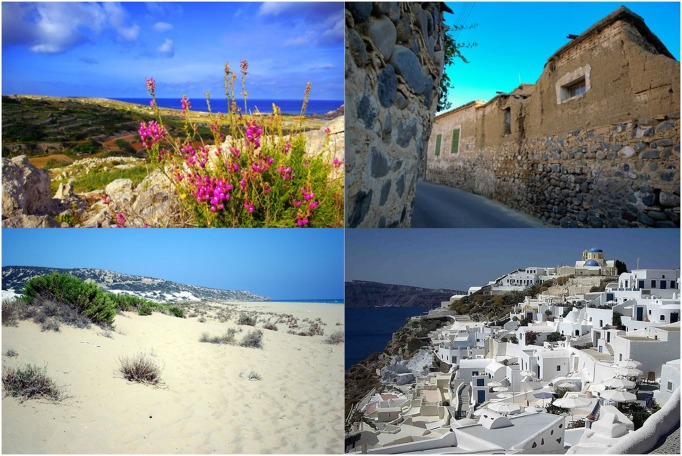
**The four Mediterranean images used for the Mediterranean *vs.* Not-Mediterranean LI-IAT**.

**FIGURE 2:**
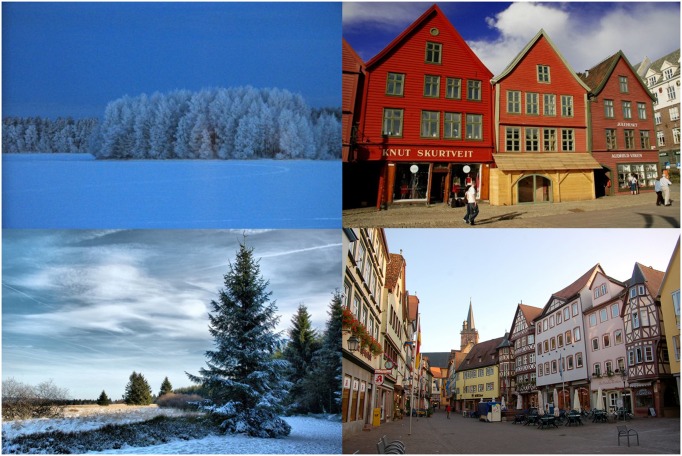
**The four Not-Mediterranean images used for the Mediterranean *vs.* Not-Mediterranean LI-IAT**.

**FIGURE 3:**
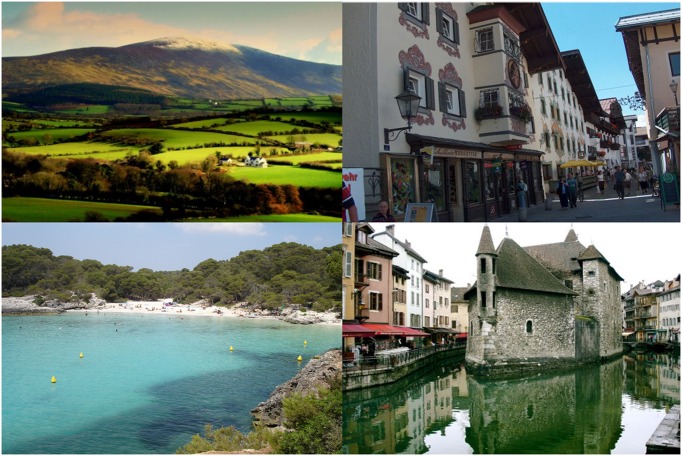
**The four European images used for the European *vs.* Not-European LI-IAT**.

**FIGURE 4:**
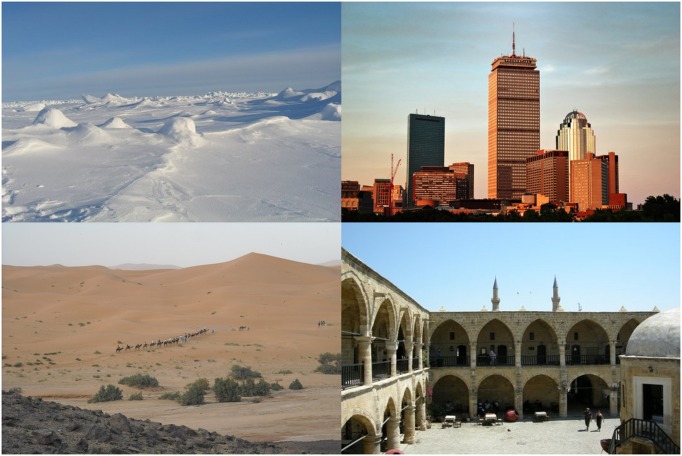
**The four Not-European images used for the European *vs.* Not-European LI-IAT**.

In order to measure the implicit level of European and Mediterranean Landscape Identity, two IATs were developed, with both single and combined categorization tasks, using for each category (Self vs. Other) four stimulus-words (used by Schultz et al., 2004; see **Table 1**) that were presented in a randomized order within each block of trials. The experimental tasks were performed by means of a 13′′ screen laptop. The set of stimuli selected for the IAT tasks included 16 landscape images, i.e., four for each target of the two IAT tasks (European vs. Not European, Mediterranean vs. Not Mediterranean), balanced for natural vs. built landscapes. For the selection of the 16 images, three steps were followed. In the first step, it was chosen a first set of 120 images, i.e., 30 images (15 of them depicting natural environments and 15 of them depicting built environments) for each of the four poles Mediterranean vs. Not Mediterranean and European vs. Not European, on the basis of their supposed fit with the targets. In the second step, a sample of two hundred European residents of different European countries, with a mean age of 32 years (*SD* = 11.72), participated to an online pilot study. They were instructed to focus on the depicted place (and not on the content of the picture, following the suggestions of Scott and Canter, 1997), and then to rate the degree to which each landscape is prototypical of European *vs*. Not European, Mediterranean *vs*. Not Mediterranean, and Natural *vs.* Built scenarios, using three 5-point semantic differential scales (i.e., ranging from, respectively, “European” to “Not European,” “Mediterranean” to “Not Mediterranean,” and “Natural” to “Built” environment). Their ratings produced the selection of the 32 most prototypical pictures, i.e., eight for each of the four poles. In the third step, three landscape architects selected the final set of 16 stimuli (four for each category, balanced for natural vs. built). The four sets of stimuli are reported from **Figures 1**–**4**.

**Table 1:** The eight stimulus-words used in the LI-IATs.

Self	Other
I	It
Me	Other
Mine	Their
Myself	Them

As described by Greenwald et al. (1998), the entire procedure consisted of seven blocks of trials: a single target categorization task (Self vs. Other; 20 trials for both LI-IATs), a single attribute categorization task (European vs. Not European for the first IAT and Mediterranean vs. Not Mediterranean for the second one; 20 trials), an initial combined categorization task (i.e., Self or European vs. Other or Not European for the first IAT; Self or Mediterranean vs. Other or Not Mediterranean for the second IAT; two sub-blocks of 20 and 40 trials, respectively), a single target categorization task reversed (e.g., Other vs. Self, 20 trials) and a second combined categorization task (i.e., Other or European vs. Self or Not European and Other or Mediterranean vs. Self or Not Mediterranean; two sub-blocks of 20 and 40 trials, respectively). The order of the two combined blocks was counterbalanced across participants (no order effects were found).

Data from the combined blocks were used to compute the value-IATs D scores, according to the built-in error penalty procedure (Greenwald et al., 2003).

### Explicit Place Identity

**FIGURE 5:**
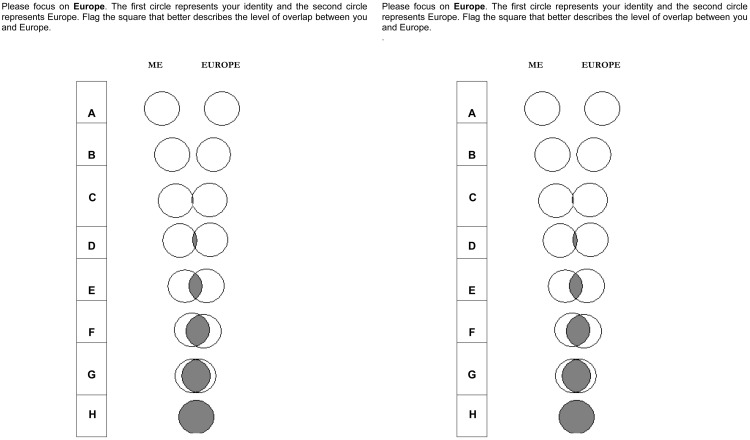
**The two graphic items measuring, respectively, European and Mediterranean explicit place identity**.

Both self-categorization items (see Bergami and Bagozzi, 2000; Schultz, 2001), adapted for measuring Mediterranean and European identification (Aron et al., 1992), are composed of eight figures, each one representing two circles in a continuum from the first figure (where the two circles are far apart) to the last figure (where the two circles are overlapped). Participants had to choose the figure that best reflects the closeness between their Self and, respectively, Europe in the first item and Mediterranean in the second item. **Figure 5** reports the two items, including the instructions for the participants.

### Pleasantness of Landscape Images

Participants rated the pleasantness of the 16 landscape pictures presented in the IATs on a five-point Likert scale, ranging from 1 (unpleasant) to 5 (pleasant). Global measures of landscape pleasantness were computed summing up European, not European, Mediterranean, and not Mediterranean scores separately. Differences between European vs. Not European and Mediterranean vs. not Mediterranean scores were computed in order to use a measure of pleasantness structurally similar to the IAT.

## Results

### Internal Consistency of LI-IATs and Descriptive Statistics

As reported in **Table 2**, both implicit and explicit measures showed skewness and kurtosis parameters included between ±1, indicating that their distribution is approximately normal. The internal consistency of the IATs was computed with a split-half estimation (Spearman–Brown corrected) using two partial D scores calculated on blocks 3–6 and 4–7, respectively. Both IATs showed an adequate level of reliability: *r*_sp_ = 0.70 for European IAT and *r*_sp_ = 0.76 for Mediterranean IAT.

**Table 2:** Descriptive statistics.

	Mean	*SD*	Skewness	Kurtosis
IAT_eu	0.74	0.38	-0.43	0.34
IAT_med	0.75	0.44	-0.70	0.66
Eu_identity	4.70	1.58	-0.53	-0.07
Med_identity	6.10	1.52	-0.70	0.18
Pleasantness_eu	-0.99	2.68	0.16	-0.19
Pleasantness_med	-0.67	1.95	-0.19	0.43

*IAT_eu, European IAT; IAT_med, Mediterranean IAT; Eu_identity, self-report measure of European identity; Med_identity, self-report measure of Mediterranean identity; Pleasantness_eu, self-report measure of European vs. not European landscapes pleasantness; Pleasantness_med, self-report measure of Mediterranean vs. not Mediterranean landscapes pleasantness*.

Landscape Identity IATs and the other measures were not significantly correlated with participants’ age. Differently, some significant gender differences emerged, with a higher mean score for males than females on both Mediterranean place identity [mean difference = -0.48; *t*(170) = -2.00, *p* < 0.05] and pleasantness attributed to the Mediterranean [mean difference = -0.34; *t*(170) = -2.27, *p* < 0.05].

Moreover, the correlations of the Mediterranean and the European LI-IATs with the corresponding place identity scales were, respectively, significant (*r* = 0.37, *p* < 0.001) and close to be significant (*r* = 0.15, *p* = 0.06). Both the LI-IATs were not correlated with the pleasantness evaluation of landscapes. The pleasantness attributed to Mediterranean landscapes was significantly correlated with Mediterranean place identity (*r* = 0.17, *p* < 0.05) while a not significant correlation emerged between the pleasantness of European landscapes and European place identity.

### Testing H1 and H2: Comparison of LI-IATs Means with the Neutral Scale Point

As expected, mean scores of both LI-IATs were positive (see **Table 1**) and significantly different from zero [*t*(171) = 22.29, *p* < 0.001 for the Mediterranean IAT; *t*(168) = 25.56, *p* < 0.001 for the European IAT] with large effect sizes (Cohen’s *D* = 1.70 for the Mediterranean IAT and Cohen’s *D* = 1.95 for the European IAT), indicating that European and Mediterranean landscapes were evaluated as more associated with the self if compared to Not European and Not Mediterranean ones. These results confirm, respectively, H1 and H2.

### Testing H3 and H4: Relationships between the LI-IATs and Explicit Place Identity

In order to further investigate the relationship between the LI-IATs and explicit place identity, two regression analyses were conducted. In the first one, the IAT measure of European place identity was included as a predictor, the self-report measure of European place identity as a criterion, and both gender and pleasantness of European landscapes as controls^1^. Similarly, in the second one, the IAT measure of Mediterranean place identity was included as a predictor, the self-report measure of Mediterranean place identity as a criterion and, again, gender and pleasantness of Mediterranean landscapes as controls. Gender and pleasantness of landscapes were included as covariates in order to control for their effect on place identity (see Discussion). Results showed a significant relationship of small size between European LI-IAT and European place identity (*R*^2^= 0.023; β = 0.15, *p* < 0.05), whereas the effects of gender and pleasantness of European landscapes were not significant. A stronger significant relationship of moderate size emerged between Mediterranean LI-IAT and Mediterranean place identity (*R*^2^ = 0.13; β = 0.36, *p* < 0.01), with significant effects of gender (β = -0.17, *p* < 0.05) and pleasantness of European landscapes (β = 0.18, *p* < 0.05). These results give a first evidence for the criterion validity of the LI-IATs.

## Discussion

This study was aimed at applying the IAT to measure European and Mediterranean landscape identity. In particular, we focused on the evaluation of the psychometric properties and criterion validity of the LI-IATs. Results showed a high split-half correlation (Spearman–Brown corrected) for both the LI-IATs, providing a first evidence for their adequate reliability. Moreover, as expected, considering that all participants are resident in geographical areas that are either European and Mediterranean, the mean *D*-scores were positive and significantly different from zero, suggesting that prototypical images of European and Mediterranean landscapes are more associated with the Self compared to, respectively, not European and not Mediterranean pictures. These results confirmed the first two hypotheses of the study, supporting the adequacy of the prototypical landscapes selected as stimuli. Finally, European and Mediterranean LI-IATs were, respectively, related to European and Mediterranean social identity, also when the pleasantness of the landscapes was controlled for. Notably, the effect size was larger for the Mediterranean LI-IAT rather than for the European one. This difference may be due to the different capacity of the European and Mediterranean landscapes to represent the corresponding categories. Indeed, since the latter area is more geographically homogeneous than the former one, the capacity of the Mediterranean landscapes to represent the corresponding area is higher with respect to the capacity of the European ones, which are much more varied and, above all, intercepts only a specific aspect of the rich and articulated meaning of the Europe notion. Overall, notwithstanding the differences found, these results support the criterion validity of both European and Mediterranean LI-IATs.

Interestingly, using the LI-IATs, other studies with experimental or longitudinal designs may be conducted in order to investigate if the specificity of the European landscapes, despite its variety, may enhance the feeling of belongingness to Europe in its citizens, as hoped by Habermas (1998). In fact, considering the significant correlations found between the LI-IATs and European and Mediterranean place identity (here conceived as “localized” social identities, see also Valera and Pol, 1994), such images are expected to convey social meanings (see Twigger-Ross et al., 2003) that go beyond the depicted scenes, including historical memories, cultural heritage, and direct and/or indirect experiences. Pictures of a recognizable European landscape may thus be able to promote a positive identification with Europe in European citizens, as found in a cross-cultural study run in diverse Italian and Spanish regions (Fornara et al., submitted). This would support a positive place identity process, as postulated by Carrus et al. (2014), and help higher community identification with the Europe concept. In other words, the feeling of “Europeanness” (Wodak, 2004) may be fostered by putting the landscape into the foreground.

Similarly, on the basis of the present results, future studies with experimental or longitudinal designs may use the LI-IAT to investigate the degree to which the Mediterranean landscapes can influence the identification with the Mediterranean. In fact, even though in this case it is not present a socio-political entity (as for Europe) that can be perceived as a reference target, the Mediterranean typical traits (see Petruzzellis and Craig, 2014) seem to be well reflected also by the landscape. It is not surprising that the internationally acknowledged beauty and pleasantness of typical Mediterranean scenes, which are commonly used to attract tourism, are elements of proudness for those who live in the Mediterranean area. For that reason, consistently with the SIT (Tajfel and Turner, 1986), such elements may represent important markers of a positive social identity related to the Mediterranean belongingness.

Even though our findings provide evidence for the adequacy of the landscapes selected and the criterion validity of the LI-IATs, some limitations of this study need to be reported.

A first limitation is the sample recruitment at national level only, that does not allow an evaluation of the LI-IATs capacity to detect possible differences across residents in different European and Mediterranean geographical areas. Thus, we cannot exclude that residents of different European countries may differ in their responses to European landscapes. In a similar vein, the identification with the Mediterranean may be less pronounced in French residents, as found by Petruzzellis and Craig (2014), and not predictable for residents in non-European countries, such as North African or Near East countries. Future cross-cultural research is thus needed in order to further validate these tools in other European and Mediterranean countries.

A methodological limit regards the measurement of the automatic level of landscape identity, that is exclusively based on the classical IAT paradigm. In future studies, it may be important to test the convergent validity of the LI-IAT developing other implicit measures of landscape identity, using for instance the Single Category IAT (Karpinski and Steinman, 2006).

A further point that needs to be mentioned concerns the partial conceptual overlap, in literature, of place-identity and other place-related constructs (see Fornara et al., 2010), such as place attachment (Altman and Low, 1992), place dependence (Stokols and Shumaker, 1981), place belongingness (Korpela, 1989), rootedness (Relph, 1976), sense of place (Hummon, 1992), and sense of community (Perkins et al., 1990), just to name a few. Nevertheless, the theoretical debate around similarities and differences among these constructs and how they are operationalized are beyond the aims of this manuscript.

## Conclusion

This study used the IAT to measure Mediterranean and European landscapes identities. Results showed that: (i) internal consistency of the two LI-IATs is adequate; (ii) participants identify themselves more with European or Mediterranean landscapes rather than with those landscapes that are, respectively, Not European or Not Mediterranean; and (iii) implicit European and Mediterranean landscape identities are related, respectively, to explicit European and Mediterranean place identities, even when pleasantness of landscapes is controlled for. These results support the validity of the LI-IATs and confirm that European and Mediterranean landscapes may be used as scenarios of identification, even though they represent larger geographical scales if compared to those usually investigated in similar studies (e.g., see Hernández et al., 2007; Lewicka, 2010).

In conclusion, this study provides a first evidence for the reliability and criterion validity of the LI-IATs, opening new ways for understanding the nature of landscape identification, along with the mechanisms that underlie its activation and development.

## Author Contributions

FF developed the research idea (together with RT), led its preparation and realization, and is the main contributor of the paper writing (in terms of both structure and content). FD participated to the preparation of the research design, made the data elaboration, and is the main contributor of the results section, playing also an important role for the writing of the other sections. RT developed the research idea (together with FF) and played an important part in the various phases of stimuli selection and in the supervision of the data collection phase. SP played an important part in the data collection and in the review of the reference literature.

## Conflict of Interest Statement

The authors declare that the research was conducted in the absence of any commercial or financial relationships that could be construed as a potential conflict of interest.

## Footnotes

1 In both regression analyses, age was not included as a covariate since it was not significantly related to both explicit place identities and LI-IATs.
